# Radar Recorded Child Vital Sign Public Dataset and Deep Learning-Based Age Group Classification Framework for Vehicular Application

**DOI:** 10.3390/s21072412

**Published:** 2021-03-31

**Authors:** Sungwon Yoo, Shahzad Ahmed, Sun Kang, Duhyun Hwang, Jungjun Lee, Jungduck Son, Sung Ho Cho

**Affiliations:** 1 Department of Electronic Engineering, Hanyang University, Seoul 04763, Korea; irishtaco@hanyang.ac.kr (S.Y.); shahzad1@hanyang.ac.kr (S.A.); sunee512@hanyang.ac.kr (S.K.); 2 Electronics Convenience Control Evaluation Team, Hyundai Motor Company, Gyeonggi 18280, Korea; dhhwang@hyundai.com (D.H.); jungjun.lee@hyundai.com (J.L.); jungduk.son@hyundai.com (J.S.)

**Keywords:** vital sign monitoring, FMCW radar, smart sensor applications, GoogLeNet, deep learning

## Abstract

The ongoing intense development of short-range radar systems and their improved capability of measuring small movements make these systems reliable solutions for the extraction of human vital signs in a contactless fashion. The continuous contactless monitoring of vital signs can be considered in a wide range of applications, such as remote healthcare solutions and context-aware smart sensor development. Currently, the provision of radar-recorded datasets of human vital signs is still an open issue. In this paper, we present a new frequency-modulated continuous wave (FMCW) radar-recorded vital sign dataset for 50 children aged less than 13 years. A clinically approved vital sign monitoring sensor was also deployed as a reference, and data from both sensors were time-synchronized. With the presented dataset, a new child age-group classification system based on GoogLeNet is proposed to develop a child safety sensor for smart vehicles. The radar-recorded vital signs of children are divided into several age groups, and the GoogLeNet framework is trained to predict the age of unknown human test subjects.

## 1. Introduction

The contactless detection and monitoring of human vital signs has shown progress in several industries, such as healthcare [1], search and rescue operations [2], and context-aware smart sensor development [3]. Recently, radar sensors have emerged as a candidate solution for simultaneously extracting vital signs such as the heart rate (HR) and breathing rate (BR) in a contactless fashion [2,4,5,6,7]. For example, Alizadeh and coworkers [8] recently used 77 GHz frequency-modulated continuous-wave (FMCW) radar to extract human vital signs in a bedroom environment. Specifically, for the case of child vital sign monitoring, Cruz et al. [9] provided a theoretical framework for in-vehicle vital sign monitoring with radar. Similarly, in-vehicle passenger detection and classification system has also been proposed [10]. After the successful extraction of BR and HR data, further analysis of these signals using deep-learning algorithms was performed in several applications, such as mental stress monitoring using electrocardiography (ECG) signals [11]. With many ongoing studies, the provision of radar-based vital sign datasets remains a challenge in the research community.

Currently, a huge attention is being paid on developing public datasets in different research areas. For example, a dataset presented by Deng and coworkers titled as ImageNet [12] has been used in over 25 thousand studies. In addition to that, Riquelme et al. [13] presented an infrared sensor dataset of fall detection. Similarly Geissinger and Asbeck [14] in 2020 recently proposed a human motion dataset using inertial sensors. Another similar activity recognition dataset was proposed in [15]. Similarly SisFall and AnkFall datasets of fall detection was proposed in [16,17] respectively. Nevertheless, for radars, few public datasets also exits and amongst them are, the two vital sign datasets presented by Shi et al. [18,19], the synthetic aperture radar (SAR) dataset provided by Wang et al. [20], and the oxford’s car robot dataset [21]. Although several studies have been conducted for vital sign detection and monitoring, public data sets are lacking. Shi and coworkers [19] provided synchronized radar-recorded human vital signs; however, all the participants were above 20 years of age, and the radar used was a single-frequency continuous-wave radar. No such vital sign dataset exists for the FMCW radar. Consequently, we provide children vital sign data with FMCW radar. With the collected data, we have performed data-validation (by comparing it with a clinical sensor,) and have suggested an additional use case application as well.

The objectives and the motivations of our work can be summarized as follows:

First, we provide a child vital sign dataset recorded from 50 children aged less than 13 years. Recruiting children and making them follow a set of protocols for data collection are difficult tasks. To the best of our knowledge, this is the first study that provides an FMCW radar-recorded dataset of child vital signs. This public repository will provide a competitive environment among the research community to test the accuracy of different (FMCW) radar based vital sign extraction algorithms. It can also be beneficial for the researchers who do not have access to the radar hardware. Additionally, this is the first public dataset that synchronizes the FMCW radar-based vital sign data with the data from a clinical reference sensor for the purpose of validation. The reference sensor used in our study is widely being used in the medical industry. We believed this dataset is very important in digital healthcare and assisted living applications.

Second, we performed vital sign extraction through FMCW radar to demonstrate the validity of presented dataset. For this purpose, the collected FMCW radar data is statistical validated against Nihon Kohden, a clinical reference sensor. Currently, not much work has been reported on children vital sign measurement and monitoring with the FMCW radar. The possible reason is the fact that gathering data from children is not easy (though, few works have been done for neonates). We present vital sign measurement of children with the FMCW radar in order to provide additional results in vital sign measurement community.

Finally, we demonstrate a deep learning-based age group classification framework as one of (the many) use case scenarios of presented dataset. With the collected data, child age-group classification is performed. This work is particularly important for future in-vehicle applications for safety and convenience. One way to ensure child safety is to ensure that the rules and regulations regarding child safety are being followed properly; notably, for different age groups, the child seat requirements are different. In addition, recently, with the revolution of smart transportation systems, vehicles should be aware of the type of passengers sitting inside. The successful identification of the age group of a child riding in vehicle can permit the smart vehicle to determine whether the installed child seat is suitable or not. Previously, attempts have been made to predict the human age-group with camera sensors [16]. Radar sensor offers several advantages over a traditional camera such as being less prone to lightning conditions [22]. Additionally, a radar sensor has no related privacy issue. As discussed earlier, in this paper, we also suggest an age group classification framework with a radar sensor in combination with a deep-learning algorithm. We aim to demonstrate (an initial study) that FMCW radar can used to develop age group classification framework as well. With the collected data, we used the GoogLeNet architecture for age group classification. We divided children into different age groups and trained the GoogLeNet architecture developed by the Google AI team [17].

In short, we aim to provide a public dataset of children vital signs recorded with FMCW. For validation of our dataset we performed the comparison between FMCW radar and clinical sensor. Additionally, a use case scenario (other than vital sign extraction) is also presented at the end.

Based on the aforementioned discussion, for children data, we present a public dataset that contains:

- Raw signals of FMCW radar, reflected from child’s chest. This raw signal contains the reflections from a human chest and all the component present within the radar’s operation range. Apart from developing the vital signs extraction algorithms, the researchers may use this data to develop or compare clutter removal techniques using the raw signals.
- The respiration and heartbeat signal from a clinically approved sensor, BSM6501K (Nihon-Kohden, Tokyo, Japan). These reference signals were synchronized with raw radar signal in the time-domain.
- The details related to the age, gender, height, and BMI of the involved human participants.
- As a first example, a MATLAB code is included in the public repository to extract the berating rate and heart rate using the raw radar signal. We included all the basic building block required to process FMCW radar signal based vital signs extraction.

The freely available data repository is accessible at FigShare [23]. While offering credits to main article, the data can freely be used in academia.

The remainder of the paper is organized as follows. Section 2 describes the methods and materials, including participants, the data collection environment, the vital sign extraction process, and the structure of the presented dataset. Next, Section 3 titled as Experimental validation section, the correlation between the radar system and the clinical reference sensor is assessed using correlation and Bland–Altman plot analyses. Section 3 additionally includes a use case scenario of the presented dataset for child age classification in vehicular applications. Section 4 concludes the paper.

## 2. Materials and Methods

### 2.1. Participants

For data acquisition, we recruited 50 children aged less than 13 years. The entire data acquisition process was performed in the presence of their parents. Prior to conducting the experiment, an informed consent signature was acquired from parents. All the experiments were conducted in according to the guidelines provided by the local ethics committee (HYUH 2017-05-004). The children participating in data collection included 24 boys and 26 girls with an average age and body mass index (BMI) of 5 and 16.68, respectively. We advertised about the project openly and recruited 50 participants.

Only those participants were invited for the data collection process whose guardians agreed with all the terms and condition of data collection process. Our aim was to have a balanced age distribution. In addition, we tried to balance the number of male and female participants.

To reduce body movements and provide a flexible data collection environment, we used car seats instead of chairs while collecting data from children under 6 years of age. Since it is difficult to make children follow a standard operating procedure (SOP), experiments were conducted under the strict supervision of expert researchers to ensure that the data collected through radar and the clinical reference sensor were similar.

The table in Appendix A represents the personal information of participants and consists of sex, birth date, weight, BMI, and height information. Data from the remaining participants have been added to FigShare (https://figshare.com/s/936cf9f0dd25296495d3 (accessed on 29 March 2021) [23] with the dataset in a folder named “Human Data”.

### 2.2. Data Collection Environment and Process

**Figure 1:**
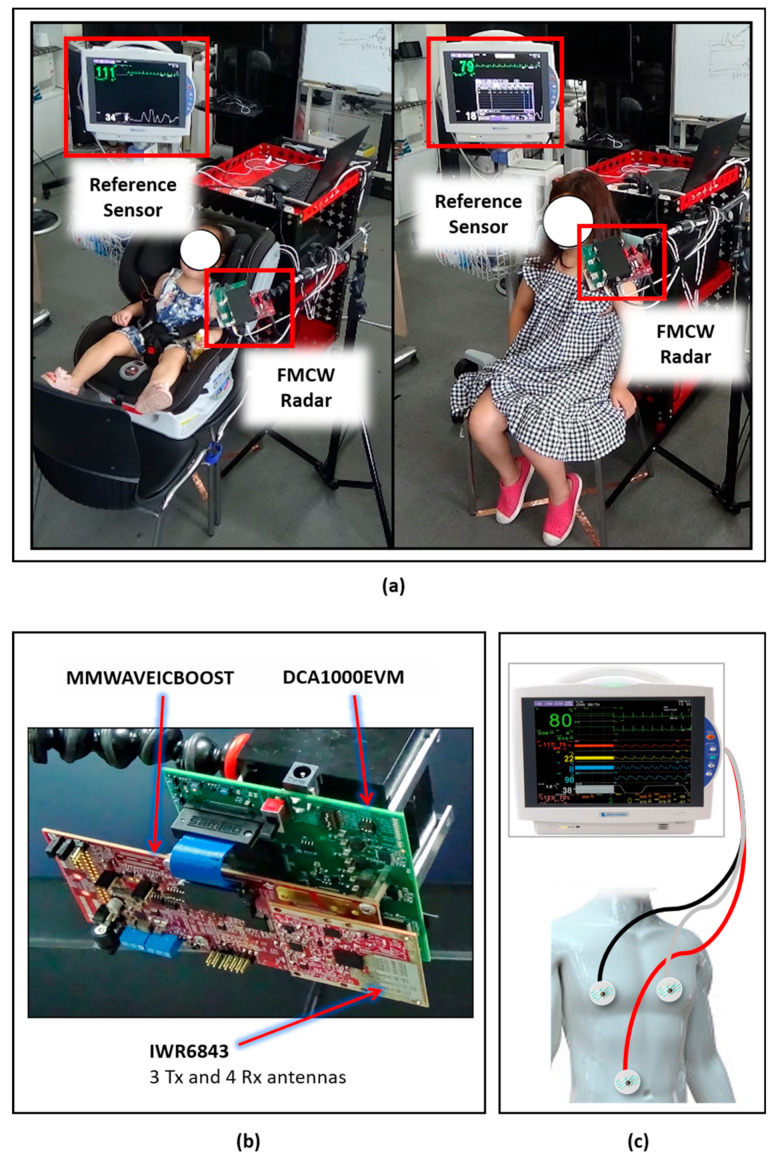
Data acquisition setup: (**a**) Data collection environment. Left: Child younger than 6 years of age sitting in a car seat. Right: Child greater than 6 years of age sitting in a normal seat. (**b**) Frequency-modulated continuous-wave (FMCW) radar system for data acquisition. (**c**) Setup and connectivity details for the clinical reference sensor.

All data collection experiments were conducted at the Fusion Technology Center (FTC) at Hanyang University, Seoul, Korea. As stated earlier, experienced researchers controlled the entire process and explained the procedure to the participants and their parents. The participants were sitting in a chair in the center of the room, and if the participant was under 6 years of age, a support (car) seat was used. Figure 1a shows the overall experimental setup for data collection, including an FMCW radar sensor (IWR-6843) that is designed and manufactured by Texas Instrument (Dallas, TX, USA), a clinical reference sensor BSM6501K manufactured by Nihon-Kohden (Tokyo, Japan), and a host computer (Intel Core i7, Intel, Santa Clara, CA, USA). The TI (Texas Instruments) FMCW radar system shown in Figure 1b was selected due to its proven effectiveness in the vital sign extraction [24]; additionally, it has been used in human sensing applications such as gait analysis [25] and hand gesture recognition [22,26].

The BSM6501K patient monitor, shown in Figure 1c, was used to compare and validate the vital signs measured by the radar system. Note that only the radar sensor was connected to the computer, and the Nihon-Kohden saves data directly in Secure Digital (SD) cards plugged into the device. Next, the usage procedures of the reference sensor and radar system are discussed in separate subsections.

### 2.3. Reference Sensor

The reference sensor, BSM6501K, shown in Figure 1c, is a clinically approved sensor that has been extensively deployed as a bedside patient monitoring device. The BSM6501K provides heart rate and respiration rate data, and the corresponding signal waveform. The heart rate is extracted using an ECG technique as explained in reference [27], and the respiration rate is extracted by utilizing the transthoracic impedance pneumography technique [28].

As shown in Figure 1c, the three electrodes colored red, black, and white were connected at three different locations on the human body; this is a standard technique for ECG signal monitoring at hospitals. Note that this sensor provides a waveform for BR and HR data collection at a rate of 125 measurements per second. Consequently, the raw waveform considered provides more samples than radar-extracted vital signs.

### 2.4. Radar Sensor

**Figure 2:**
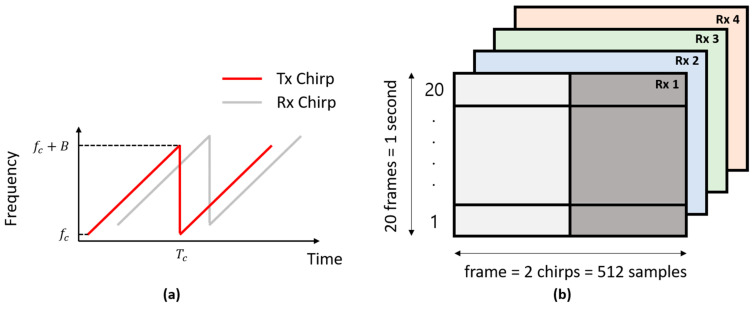
(**a**) The transmitted and received chirps and (**b**) the received radar data signals for all the four receivers, denoted as *Rx* 1, *Rx* 2, *Rx* 3, and *Rx* 4.

We used FMCW radar for vital sign extraction, as shown in Figure 1b. While acquiring the data, an FPGA module called DCA1000 and a carrier card called MMWAVEICBOOST formed a gateway between the IWR-6843 FMCW radar and the host computer. The FMCW radar transmits a periodic signal $x_{T}\left(t\right)$ with a linearly increasing frequency, known as a chirp signal. Figure 2a shows the chirps used in our experiments. The transmitted signal can be expressed as follows [29]:

(1) $$x_{T}\left(t\right)=A_{T}\cos\left(2\pi f_{c}\left(t\right)+\;\pi \frac{B}{T_{c}}t^{2}+\phi \left(t\right)\right)$$

where $A_{T}$ denotes the transmitted power of $x_{T}\left(t\right)$ and $T_{c}$, $f_{c},$ and B represent the duration, starting frequency, and bandwidth of the chirp, respectively. The term $\phi \left(t\right)$ represents the phase of $x_{T}\left(t\right)\;$at time t.

The hardware specifications and technical parameters of the FMCW radar used are listed in Table 1. As shown in Table 1, the radar sensor IWR-6843 had three transmitters and four receivers. Additionally, it has a starting frequency of 60 GHz and spans up to 64 GHz. In our experiment, we used the customized setting shown in Table 2 based on vital sign requirements. Since vital signs can be measured with few chirps, we used only 2, but a higher number of chirps is required for detecting multiple targets. As stated in Table 2, we used 1 transmitter (*Tx*) and 4 receivers (*Rx*) at 20 frames per second (FPS). The resulting radar data cube for 1 s of vital sign information is shown in Figure 2b.

Here, the radar data matrix had a size of 512 × 20 × 4, corresponding to 512 samples per frame, 20 frames per second and 4 *Rx* devices. The horizontal axis in Figure 2b shows the samples transmitted in a single frame, and the vertical axis shows the number of transmitted frames. Here, 20 frames constitute a time duration of 1 s.

Upon reflection from a target present within the operation range of the radar, the reflected signal $x_{R}\left(t\right)$ at the receiver can be expressed as

(2) $$x_{R}\left(t\right)=\alpha A_{T}\cos\left(2\pi f_{c}\left(t-t_{d}\right)+\pi \frac{B}{T_{c}}{\left(t-t_{d}\right)}^{2}+\phi \left(t-t_{d}\right)\right),$$

where α and $t_{d}$ denote the attenuation and time delay, respectively. Note that the target is the chest vibration of a child sitting in front of the radar. The signal in Equation (2) is the delayed and attenuated version of the transmitted signal in Equation (1).

**Figure 3:**
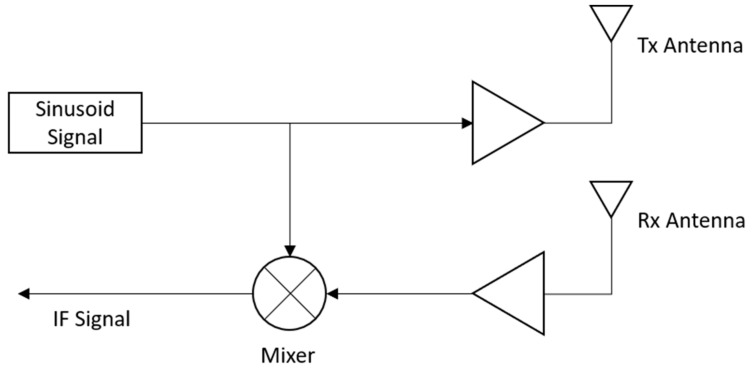
Processing of received FMCW radar signals for extracting the intermediate frequency (IF) signals.

Figure 3 shows the processing steps for the received radar signal $x_{R}\left(t\right)$ presented in Equation (2). As shown in Figure 3, the received signal is multiplied by the transmitted signal using an analog mixer, and the resulting product signal is termed the intermediate frequency (IF) signal. The IF signal $x_{IF}\left(t\right)$ can be expressed as a complex exponential signal consisting of in-phase and quadrature signals, as shown in Equation (3).

(3) $$x_{IF}\left(t\right)=A_{IF}e^{j\left(2\pi \frac{B}{T_{c}}t_{d}t+2\pi f_{c}t_{d}+\pi \frac{B}{T_{c}}t_{d}^{2}+\phi \left(t\right)-\phi \left(t-t_{d}\right)\right)}$$

In Equation (3), $A_{IF}$ represents the signal power of $x_{IF}\left(t\right)$. Since we are deploying the radar system in a short range, the term $\pi \frac{B}{T_{c}}t_{d}^{2}+\phi \left(t\right)-\phi \left(t-t_{d}\right)$ can be ignored, resulting in the following simplified form of the above equation.

(4) $$x_{IF}\left(t\right)=A_{IF}e^{j\left(2\pi \frac{B}{T_{c}}t_{d}t+2\pi f_{c}t_{d}\right))}\;\;$$

The raw data provided in our data repository at FigShare [23] include the IF signals represented in Equation (4).

### 2.5. Radar Signal Processing for Vital Sign Extraction

**Figure 4:**
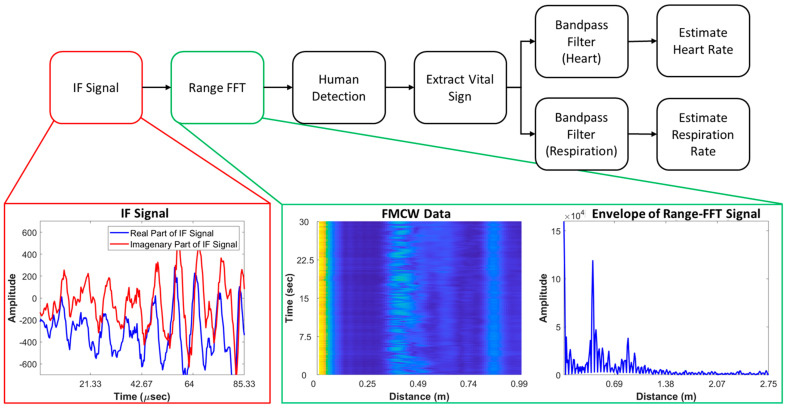
Process of heart rate (HR) and breathing rate (BR) extraction using the IF signals of radar and intermediate outputs.

The process for vital sign extraction with the FMCW radar is shown in Figure 4. According to Equation (4), upon reflection from a child’s chest, the signal distance will change with respect to time. This change is expressed as the frequency difference between the transmitted and received signals. With the range-fast Fourier transform (range-FFT) approach, the change in distance can be extracted. In this paper, vital signals were detected and extracted by observing this change in distance. In specific, the presented human detection block in the proposed algorithm tries to locate the distance where the human is located. The output of range-FFT is a 2-D matrix and within this 2-D range-FFT matrix, the distance where the highest value of variance was observed is considered as the vibration point from the child’s heartbeat and breath. The vital sign signal information is gathered by accumulating the values located at the distance corresponding to maxima of variance. As shown in Figure 4, we used two separate bandpass filters for HR and BR extraction. As stated earlier, children did not follow SOPs well, and frequent and random body movements occurred while collecting the data. In our case, we extracted the HRs and BRs of all 50 participants with the observations given in Table A1. With our dataset, the research community could potentially design more sophisticated approaches to overcome difficult issues such as human body movement during vital sign measurement.

The extracted vital signs of radar and the reference signals are presented in the data validation Section 3.

### 2.6. Data Records

**Figure 5:**
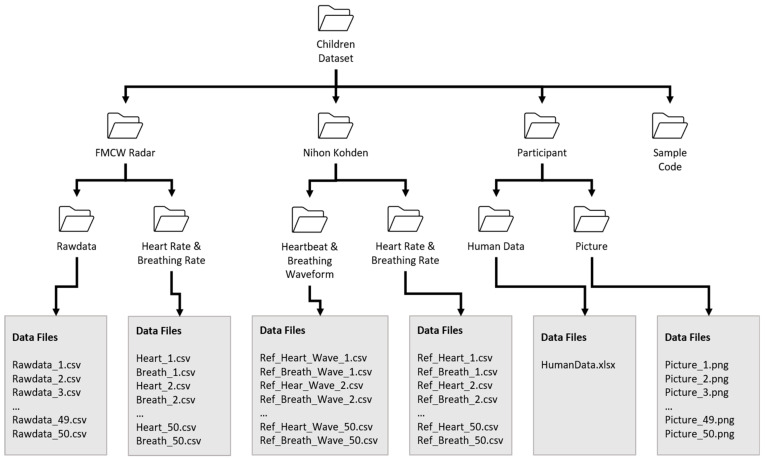
The structure of the proposed child vital sign dataset.

Figure 5 shows the structure of the presented dataset. The main dataset contains three folders containing FMCW radar data, clinical reference sensor (Nihon Kohden) information and the personal information of participants. Participants’ personal information is included separately in the folder named “Participants”. As shown in Figure 5, we include both the raw data and the extracted vital sign data in separate repositories. Here, the folder named “Rawdata” contains the extracted IF signals, as outlined in Figure 3. Additionally, the folder named “Vital sign” contains the vital signs extracted using the algorithm presented in Figure 4. Each of the subfolders also contains 50 files corresponding to each individual participant. The details regarding the structure and the contents of each individual file are included within each folder. Additionally, we added a MATLAB code that can be used to access and observe the vital signs of each individual participant.

## 3. Experimental/Data Validation and Use-Case Application

### 3.1. Sensor Synchronization

The recorded signals from both sensors were synchronized in the time domain before technical validation. Additionally, the uploaded data from the radar system and reference sensor were precisely synchronized. Since the children did not follow the SOPs, each individual recording was thoroughly inspected, and if severe quality degradation was noted, the data were collected again.

### 3.2. Correlation Between the Clinical Sensor and FMCW Radar Datasets

**Figure 6:**
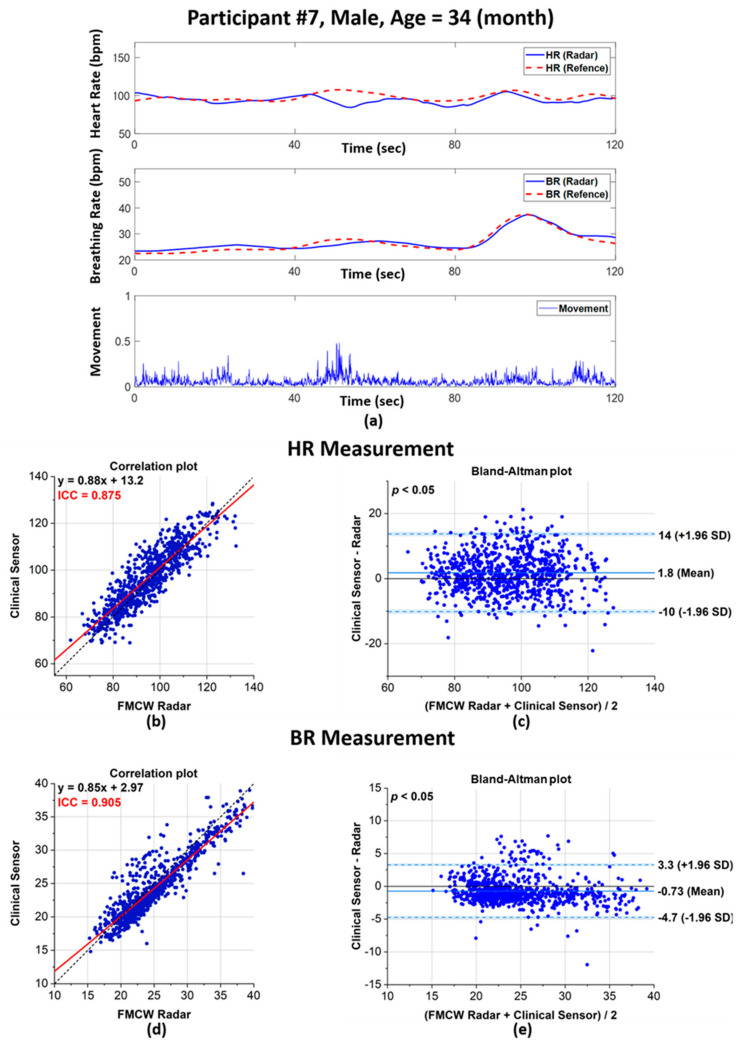
Performance evaluation of the FMCW radar system and clinical sensor for vital signs extracted from human volunteers: (**a**) HR and BR rate comparison between the radar system and clinical sensor with the calculated body movements for human volunteer 7; (**b**) scatter plot of the extracted HRs; (**c**) Bland–Altman plot of the extracted HRs; (**d**) scatter plot of the extracted BRs; and (**e**) corresponding Bland–Altman plot for the extracted BRs.

To validate our data, we performed a correlation of the vital sign data extracted with the radar sensor and the reference clinical sensor. Figure 6 shows the comparison of vital sign measurements obtained with the FMCW radar system and the reference sensor. As an example, Figure 6a shows the HR and BR measurements for one human volunteer, and the scatter plots and Bland–Altman plots in Figure 6b–e shows the validation of the overall HR and BR measurements. The HR and BR of human volunteer No. 7 are presented in Figure 6a, which shows that the patterns of both sensors were similar. In addition, the third row of Figure 6a is the body movement index. As stated in the above Section 2, vital signs should be measured only when a participant’s body was at rest, and the peak in the graph reflected an instance when body movement occurred.

As represented in Figure 6b,d, the extracted HRs and BRs show a high intraclass correlation (ICC) between the radar system and clinical sensor. A previous study [30] reported that an ICC between 0.7 and 1 corresponds to high reliability and a high correlation between two quantities.

For our dataset, the ICC values for the HR and BR were 0.875 and 0.905, respectively. Additionally, the red line in Figure 6 shows the regression analysis results for both quantities; this line plots close to the expected values, plotted as a dotted black line.

Bland–Altman plots can effectively reflect the agreement or disagreement between two different quantities. In Figure 6c,e, the horizontal axis corresponded to the average values of both the clinical sensor and radar, whereas the vertical axis corresponded to the difference between the values. Here, the dotted lines show the limit of agreement, commonly known as the LoA. For our dataset, very small biases were observed for the HR and BR at 1.8 and −0.73 beats per minute (bpm), respectively. Additionally, the LoAs for the HR and BR are −10–14 bpm and −4.7–3.3 bpm, respectively. There was no proportional bias in the HR and BR measurements, but the width of the LoAs was narrower for the BR than for the HR, which suggests that BR measurements were more precise than HR measurements. To ensure that there was no significant difference between the radar and reference sensor, the *p*-value from the Kruskal–Wallis *t*-test was observed. A *p*-value < 0.05 was observed, which indicates that there was no significant difference between the measurements from the radar and the reference sensor. The *p*-values for the HR and BR measurements were 0.003 and 0.001, respectively.

### 3.3. Demonstration of GoogLeNet-Based Age Group Classifier

The main focus of the presented dataset is to provide a competitive platform for developing new techniques for the extraction and continuous monitoring of vital signs using the FMCW radar. In addition, here, we state another potential application of developing an inhabitant-aware sensor using the presented dataset. This experiment demonstrates the usefulness of the collected dataset for healthcare and assisted smart living sensor development. We divided the dataset into several different age groups and trained the GoogLeNet classifier [31]. Thirty seconds of data from each participant were transformed into a single-range FFT image that served as an input to GoogLeNet. In general, the 2-D range-FFT image of the received radar signal contains huge amount of information related to the target. For the case of human subject as a target, this information may contain the details related to the radar cross section (RCS) of the human body, breathing pattern, heartbeat signal, micromotions, and so on. As a result, the 2-D range-FFT image can be exploited to train deep learning models to categorize the radar-recorded data. As stated earlier, in this research, we applied a deep learning model named as the GoogLeNet classifier and has previously been used for image classification. The core of the GoogLeNet classifier is based on the convolution operations. Inspired by the visual cortex of animals, the CNNs works on the grid data (2D/3D images). A typical CNN networks is a layered network consisting of three main layers: 1) input layer, the hidden layer ant the classification layer [14]. A hidden layer in CNN further comprise of a convolution layer, the max-pooling layer, and batch normalization layer. Specifically, convolutional layer convolves the input with a fixed filter known as the kernel. A deep network is formed by stacking the layers in series to extract more rich features from the input data. For instance, in any layered CNN architecture, the input to the successive hidden layer is normally the output of the current hidden layer. Traditionally, only stacking layers on top of each other was considered as a way of increasing the accuracy of a CNN based classifier. The in-depth structural amendments (such as concatenating the output of several convolutional layers together as input to next hidden layer) were not considered. However, later several networks were prepared, which incorporated structural amendments as well. GoogLeNet presented by Szegedy and coworkers [16] used non-conventional scheme to form a complex deep network. Rather than combining the hidden layers in series, a new block named as “inception module” was introduced in the CNN model. An inception module contains several different convolution kernels at each hidden layer and the output from each layer is concatenated together that serves as an input to the next layer.

**Figure 7:**
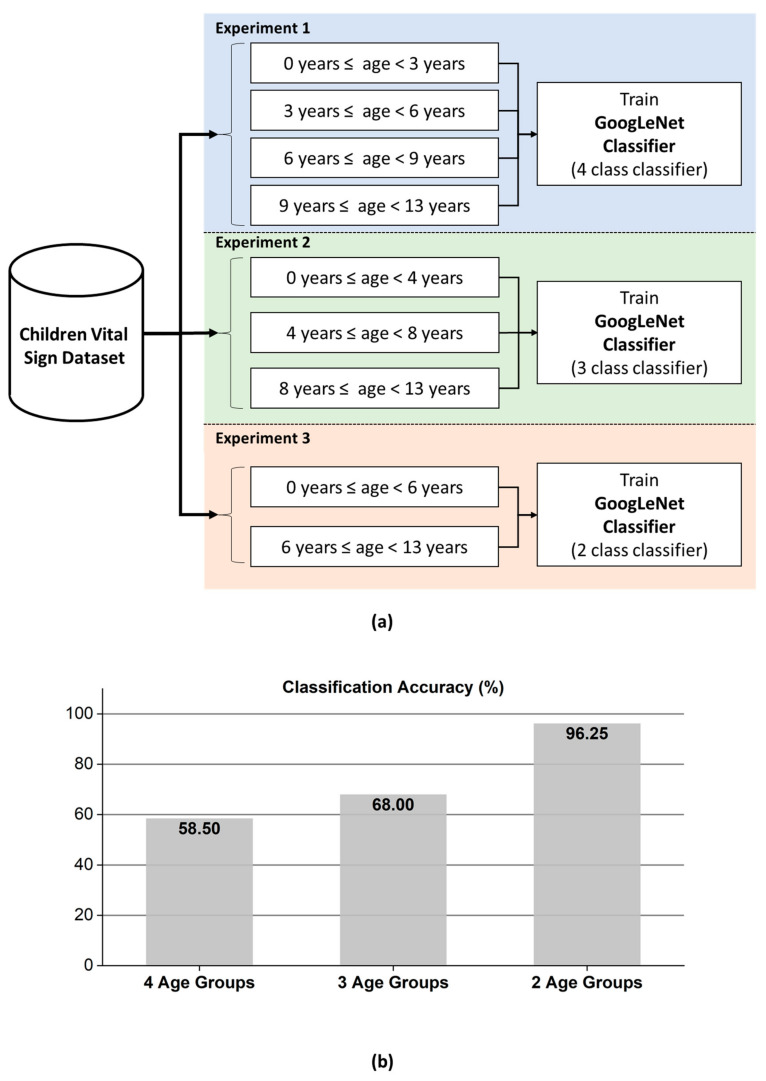
(**a**) Age-group classification experiments performed with GoogLeNet. Upper: age prediction based on four, three, and two age groups and (**b**) bar chart showing the increase in prediction accuracy with a decreased number of classes.

Rather than randomly splitting the entire dataset into a training set and a test set, we performed training on the data for 40 participants and used the data for the remaining 10 participants for evaluation. These 10 participants were still selected randomly without any choice-preferences. In addition to that, the number of participants in each age-group were not equal. Manual split was opted in order to have a balanced ration between the training and test data in each age-group. With a dataset having unequal number of samples in each class, random split might have created a biasness in the evaluation process. As shown in Figure 7, we performed three different age group classifications with the presented dataset. In the first experiment, the child vital signs were divided into four age groups: 0 to less than 3 years, 3 to less than 6 years, 6 to less than 9 years, and 9 to 13 years of age. In the second experiment, the children were divided into three age groups: 0 to less than 4 years, 4 to less than 8 years, and 8 to 13 years. Finally, in the 3rd experiment, two groups were established: 0–6 years and 6–13 years. Please note that the test data (which was intentionally separated from the training data) contains total 10 participants.

According to the classification accuracy presented in Figure 7b, the classifier for the FMCW radar system based on GoogLeNet can effectively divide children into two classes with a significantly high accuracy of approximately 96.25%. For a smart car, an FMCW radar-based vital sign sensor can be used to predict whether a seated child was less than or greater than 6 years of age with the accuracy of 96.25%. In the remaining two experiments, the accuracy was lower than that in the first experiment. Additionally, as the number of age groups decreased, increased accuracy can be achieved.

**Figure 8:**
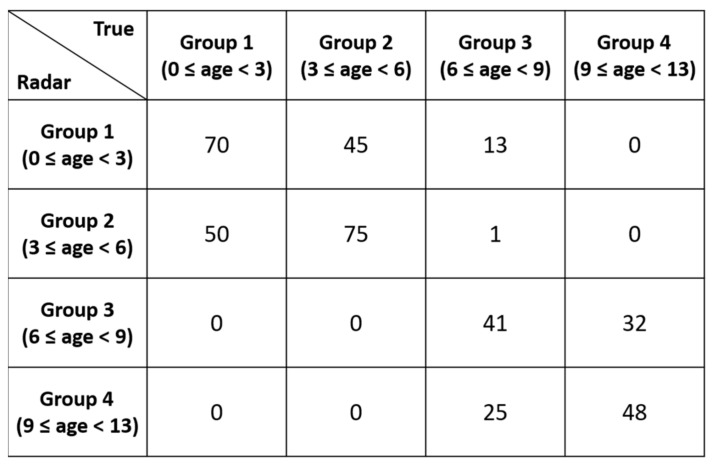
Confusion matrix of the 4 age-group classification.

**Figure 9:**
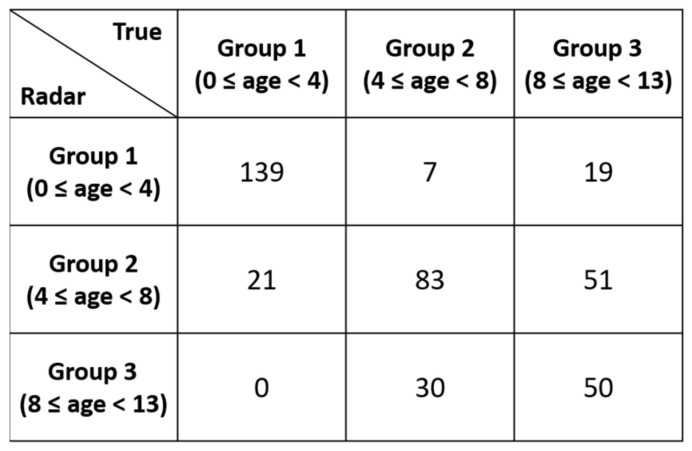
Confusion matrix of the 3 age-group classification.

**Figure 10:**
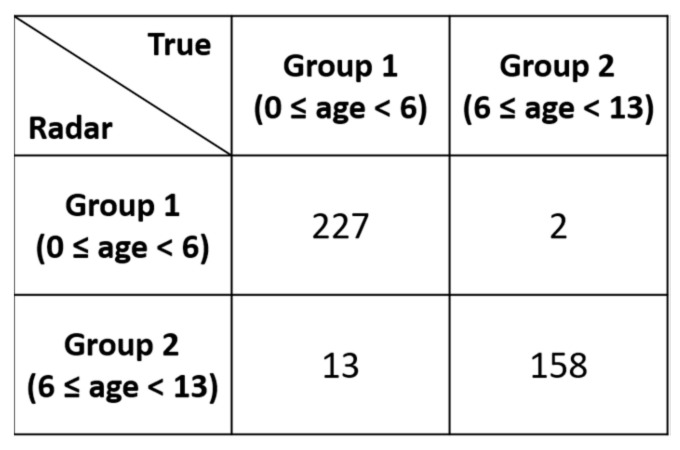
Confusion matrix of the 2 age-group classification.

As shown in Figure 7b, the two-class classifier displayed significantly higher accuracy than the three-class classifier. Figure 8, Figure 9 and Figure 10 present the detailed confusion matrixes of all three experiments shown in Figure 7a.

## 4. Conclusions

In this paper, we presented a public dataset of children vital signs using the FMCW radar. A clinical reference sensor was deployed for validating the collected radar data. Since the sensitive topic of collecting vital signs from minors (children) was covered in this research, an ethical approval and informed consent form was signed. Making children to follow experimental protocol was tedious work as a result, we observed several body movements (as shown in Figure 6a) during the data capturing process. After capturing data, an algorithm to extract vital signs is presented, which show acceptable ICC between the radar and the clinical sensor. A use case scenario of GoogLeNet based classifier demonstrated that a deep learning algorithm could be trained to learn different age-groups for their automatic classification. These classification results can consequently be considered to make a context aware sensor in smart environments such as a smart car where a car will be aware of inhabitant and can behave accordingly.

## Figures and Tables

**Table 1:** Technical specifications of the IWR-6843 TI FMCW radar.

Parameter	Value
Number of Transmit Antennas	3
Number of Receive Antennas	4
Starting Frequency ($f_{c}$)	60 GHz
Bandwidth (B)	4 GHz
*T_x_* Power	12 dBm
*R_x_* Noise	12 dB

**Table 2:** Settings of the FMCW radar for vital sign extraction.

Parameter	Value
Number of Transmit Antennas	1
Number of Receive Antennas	4
Starting Frequency ($f_{c}$)	60.25 GHz
Bandwidth (B)	3.75 GHz
ADC Sampling Rate ($F_{s}$)	3 Msps
Chirp Duration ($T_{c}$)	91.72 μsec
Number of Chirps per Frame	2
Frames per Second	20
Range Resolution ($d_{res}$)	4 cm
Maximum Range ($d_{max}$)	11 m

## Data Availability

The data presented in this study are openly available in FigShare at https://doi.org/10.6084/m9.figshare.13515977.v1, [23].

## Appendix A. Human Data for 50 Children

**Table A1:** 50 involved children in data acquisition process. Column 8 and 9 represent the beats per minute (bpm) range of each participant obtained from Nihon Kohden.

Index	Gender	Age (Month)	Height (cm)	Weight (kg)	BMI (m/kg^2^)	Car Seat	Breath Rate Range (bpm)	Heart Rate Range (bpm)
1	Female	38	97.5	16.3	17.15	Yes	16–37	83–117
2	Male	44	101.1	16.2	15.85	Yes	19–44	96–125
3	Female	15	77.5	8.7	14.48	Yes	26–56	117–150
4	Male	54	102.0	16.6	15.96	Yes	18–26	102–124
5	Female	95	125.9	28.8	18.17	No	13–39	83–112
6	Female	60	102.3	14.7	14.05	Yes	15–29	86–119
7	Male	34	101.0	14.7	14.41	Yes	19–39	89–110
8	Female	25	91.3	13.2	15.84	Yes	21–38	95–131
9	Male	57	107.7	19.3	16.64	Yes	15–27	82–107
10	Male	82	127.0	22.7	14.07	No	20–31	80–100
11	Male	117	145.7	48.8	22.99	No	22–34	81–105
12	Female	82	116.8	17.4	12.75	No	19–28	76–92
13	Female	95	125.6	23.0	14.58	No	17–24	82–99
14	Female	116	137.1	36.2	19.26	No	14–23	71–83
15	Female	53	102.4	15.2	14.50	Yes	19–34	90–107
16	Female	95	131.9	33.1	19.03	No	9–25	76–91
17	Female	75	125.2	33.1	21.12	No	15–26	77–90
18	Female	22	88.2	12.7	16.33	Yes	18–35	101–130
19	Male	42	104.5	15.1	13.83	Yes	15–39	106–128
20	Male	69	115.7	19.9	14.87	Yes	17–25	91–117
21	Male	112	127.0	26.8	16.62	No	17–28	94–120
22	Male	82	126.4	35.0	21.91	No	21–42	103–117
23	Female	84	125.0	36.6	23.42	No	13–31	74–95
24	Male	75	121.0	23.0	15.71	No	15–31	72–92
25	Male	21	84.2	12.0	16.93	Yes	19–37	95–119
26	Male	31	89.1	11.5	14.49	Yes	14–35	89–110
27	Male	31	93.0	14.4	16.65	Yes	21–37	96–128
28	Female	32	92.4	14.5	16.98	Yes	16–28	100–120
29	Male	53	104.8	16.4	14.93	Yes	16–39	88–108
30	Female	35	90.5	13.9	16.97	Yes	19–27	87–111
31	Female	63	105.4	15.1	13.59	Yes	18–24	81–106
32	Female	66	116.2	18.3	13.55	Yes	13–26	83–109
33	Female	13	78.4	9.6	15.62	Yes	24–33	106–119
34	Male	9	73.1	9.6	17.97	Yes	19–47	96–124
35	Male	112	147.0	47.5	21.98	No	13–22	79–96
36	Male	112	148.5	31.9	14.47	No	11–28	80–101
37	Male	121	144.8	46.8	22.32	No	13–31	90–115
38	Male	109	132.6	29.1	16.55	No	12–31	87–102
39	Male	124	153.1	44.1	18.81	No	15–31	70–83
40	Female	29	90.8	13.5	16.37	Yes	27–36	95–116
41	Female	44	92.0	13.5	15.95	Yes	19–39	102–120
42	Female	47	102.3	16.5	15.77	Yes	20–27	102–135
43	Female	9	69.1	8.4	17.59	Yes	26–40	115–133
44	Male	40	100.0	14.5	14.50	Yes	13–24	93–116
45	Female	7	73.2	9.6	17.92	Yes	15–24	94–112
46	Female	42	95.4	13.2	14.50	Yes	13–26	81–111
47	Female	9	74.0	9.3	16.98	Yes	17–24	103–119
48	Male	25	92.1	11.2	13.20	Yes	12–24	94–115
49	Female	148	160.0	54.0	21.09	No	18–27	76–90
50	Male	103	144.4	30.4	14.58	No	16–24	66–93
